# Postnatal Growth of Moroccan Preterm Infants: Determinants of Incomplete Catch-up Growth and Z-Score Trajectories in a Middle-Income Country

**DOI:** 10.34172/jrhs.9203

**Published:** 2025-09-15

**Authors:** Latifa Mochhoury, Khaddouj Elgoundali, Milouda Chebabe, Kawtar Chafik, Mohamed Chahboune, Amina Barkat

**Affiliations:** ^1^Higher Institute of Health Sciences, Hassan First University of Settat, Settat, Morocco; ^2^Higher Institute of Nursing Professions and Health Techniques (ISPITS), Ministry of Health and Social Protection, Rabat, Morocco; ^3^Faculty of Medicine and Pharmacy, Mohammed V. University, Rabat, Morocco

**Keywords:** Infant, Premature, Fetal growth retardation, Nutritional status, Growth and development, Breastfeeding

## Abstract

**Background:**

Prematurity and neonatal hypotrophy (defined as a Z-score below -2 for weight, length, or head circumference) increase the risk of perinatal morbidity, mortality, and long-term developmental disorders. This study examines the growth trajectories of Moroccan preterm infants and investigates the factors influencing their overall growth outcomes at six months, including weight, length, and head circumference.

**Study Design:**

A retrospective longitudinal cohort study.

**Methods:**

This study was conducted at the National Reference Center for Neonatology and Nutrition in Rabat from April to October 2023. It included 686 premature newborns (24–36 weeks) hospitalized for≥48 hours, with complete anthropometric data and follow-up of six months. Exclusion criteria were major malformations, chromosomal abnormalities, metabolic disorders, and incomplete data. ANOVA and multivariate logistic regression identified independent predictors of weight growth outcomes at six months (WAZ≥-2), adjusting for confounders (gestational age, gender, hospitalization, multiparity, phototherapy, antibiotics, and early food diversification). Results are reported as odds ratios (ORs) with 95% confidence intervals (CI). Growth curves were generated with Python. Significance was set at *P*<0.05.

**Results:**

Gestational age of≥32 weeks (OR=6.66, 95% CI: 1.21, 36.72; *P*=0.029) and multiparity (OR=12.09, 95% CI: 2.12, 68.93; *P*=0.005) predicted growth outcomes, while a hospital stay of≥10 days reduced the likelihood (OR=0.05, 95% CI: 0.01, 0.27; *P*=0.001). Male gender and antibiotic use showed non-significant trends (*P*=0.053).

**Conclusion:**

Close monitoring and targeted nutritional strategies are essential to improve postnatal growth in preterm infants.

## Background

 Prematurity constitutes a major global public health challenge, given the particular vulnerability of this population facing both physiological and anthropometric immaturity. This vulnerability is especially pronounced in hypotrophic newborns, defined by intrauterine growth restriction (IUGR) or a Z-score of < -2, which presents the highest risk of postnatal growth delay and perinatal complications.^1^

 Careful monitoring of growth patterns in these infants is essential,^2^ Z-scores for weight (WAZ), length (HAZ), and head circumference (HCZ) are key indicators of future health and development in preterm infants. Catch-up growth patterns are highly variable in this population. Rapid catch-up growth has been associated with an increased risk of metabolic syndrome and cardiovascular disease in adulthood,^3^ while failure to catch up can negatively impact overall development.^4^

 The literature reports that multiple factors influence catch-up growth in hypotrophic preterm infants, including birth weight, gestational age, type of neonatal nutrition, and early interventions such as nutritional supplementation and exclusive breastfeeding.^5^

 Despite advances in neonatal care, postnatal growth trajectories in hypotrophic preterm infants remain heterogeneous, and the determinants of catch-up growth, particularly in low- and middle-income countries, are still poorly documented.^6^

 In Morocco, as in other middle-income countries, data on postnatal growth trajectories of preterm infants are scarce, especially regarding the catch-up process in hypotrophic newborns. Although neonatal care has improved, the long-term nutritional outcomes of this vulnerable population are not systematically assessed.

 Given the increasing survival of preterm newborns, especially those with IUGR, it is crucial to understand the determinants of growth failure and incomplete catch-up in this population. This study aims to fill this gap by analyzing Z-score trajectories for WAZ, HAZ, and HCZ and to identify clinical and nutritional factors associated with catch-up growth from birth to six months. By calculating Z-scores at birth, 10 days, 1 month, 3 months, and 6 months, based on the 2013 Fenton growth charts.^7^

## Materials and Methods

###  Study design

 This retrospective longitudinal study utilized clinical data from premature newborns admitted to the National Reference Center for Neonatology and Nutrition in Rabat. The retrospective design allows the use of pre-recorded data in the hospital information system, reducing costs and workload. Data were collected from April 1 to October 31, 2023, from computerized medical records. The analysis focused on growth parameters tracked over six months post-birth.

###  Population and participant selection

 The study included a cohort of 686 premature newborns (24-36 weeks of gestation) admitted to the center.

 Inclusion criteria were:

- Neonatal hospitalization of ≥ 48 hours for a complete initial clinical assessment.
- Availability of neonatal anthropometric parameters (WAZ, HAZ, HCZ).
- Documented postnatal follow-up up to 6 months.

Exclusion criteria included: (a) major congenital malformations, (b) identified chromosomal abnormalities, (c) inborn errors of metabolism, and (d) incomplete clinical data. This strict selection ensured a homogeneous study population for analyzing early growth parameters in premature infants.

###  Sampling process

 This study applied an exhaustive retrospective sampling of all preterm newborns admitted to the National Reference Center for Neonatology and Nutrition in Rabat between April and October 2023. The initial cohort included 985 newborns. After excluding 249 infants (due to congenital malformations, genetic syndromes, or missing anthropometric data), 736 infants remained for preliminary assessment. During data screening, we identified and excluded 50 full-term infants ( ≥ 37 weeks of gestation) as they did not meet our preterm inclusion criteria. The final analysis included 686 preterm infants ( < 37 weeks of gestation), comprising:

- 72 hypotrophic infants (10.5%, defined as Z-score of < -2 using the 2013 Fenton growth charts^7^
- 614 infants with normal growth (89.5%).

###  Data collection and management

 The data were collected exclusively from computerized medical records in the hospital information system. Only records containing complete and consistent data (WAZ, HAZ, and HCZ) were included in the analysis, while records with missing information were excluded to ensure the quality of the data used.

 Anthropometric parameters, including WAZ, HAZ, and HCZ, were measured following a standardized protocol in accordance with WHO guidelines.^8^ All measurements were performed by qualified personnel, including neonatal nurses and trained pediatric residents. Weight was measured after a 30-minute fasting period using a calibrated electronic scale, length was measured using a rigid infantometer, and HCZ was assessed with a non-elastic measuring tape. Measurements were conducted at five key time points: birth, 10 days, 1 month, 3 months, and 6 months.

###  Definition and Calculation of Z-scores

 Z-scores for WAZ, HAZ, and HCZ were calculated using the 2013 Fenton growth curves, recognized for growth assessment of preterm neonates. All Z-scores were obtained from the automated calculator based on the Fenton curves, available on the University of Calgary website: https://ucalgary.ca/resource/preterm-growth-calculator. This calculator generates Z-scores normalized for corrected gestational age and gender, at different follow-up times (birth, 10 days, 1 month, 3 months, and 6 months).

 Growth categorization was based on WHO and Fenton 2013 Z-score thresholds: Z-scores between –2 and + 2 were considered normal, values below –2 indicated growth restriction (e.g., hypotrophy, stunting, or microcephaly depending on the parameter), and values above + 2 reflected excessive growth or macrosomia.

###  Statistical analysis

 Descriptive and inferential analyses were conducted to explore growth trajectories and identify predictors of weight catch-up. Data were analyzed using SPSS version 26. Descriptive statistics included means, standard deviations, and proportions. Between-group comparisons (hypotrophic vs. non-hypotrophic infants) were performed using the chi-square test, Fisher’s exact test, and McNemar test for paired categorical data.

 To analyze the anthropometric Z-scores (WAZ, HAZ, HCZ) across five time points (birth, 10 days, 1 month, 3 months, and 6 months), repeated-measures ANOVA was employed to analyze longitudinal changes in Z-scores over five time points (birth, day 10, 1 month, 3 months, and 6 months), as this method is appropriate for within-subject comparisons across multiple time points.

 Although more advanced models such as mixed-effects models were considered, repeated-measures ANOVA was chosen for its interpretability and adequacy in the context of a fixed cohort with complete follow-up data. To ensure validity, the assumptions of ANOVA were systematically checked: normality of residuals was verified using the Shapiro-Wilk test, and sphericity was assessed using Mauchly’s test. When the assumption of sphericity was violated, the Greenhouse-Geisser correction was applied. These methodological precautions support the robustness of the statistical inferences derived from the longitudinal data.

 Binary logistic regression models were used to examine associations between selected perinatal and clinical variables and the likelihood of achieving weight catch-up (WAZ ≥ –2) at 6 months in hypotrophic infants. Each variable was assessed separately in univariate models based on its clinical relevance and data availability. Odds ratios (ORs) with 95% confidence intervals (CIs) were reported. No multivariate adjustment was performed.

 Growth curves were plotted using Python (Matplotlib and NumPy libraries).

 All tests were two-sided, and a *P* < 0.05 was considered statistically significant. The study adhered to the STROBE guidelines for reporting observational studies.

 Missing data were handled using case-wise deletion; therefore, no imputation methods were applied due to the retrospective design of the study.

## Results

###  Maternal and neonatal characteristics 

 Among the 686 preterm infants, 72 (10.5%) presented with hypotrophy, defined as a Z-score < –2, compared to 614 (89.5%) with normal growth. The mean maternal age was 26.42 ± 3.37 years. The percentage of mothers aged ≥ 35 years was higher in hypotrophic newborns (8.8% vs. 4.0%), although this difference was not statistically significant (*P* = 0.072). No significant associations were found with socioeconomic status (*P* = 0.805) or maternal education level (45.0% vs. 38.0%; *P* = 0.156).

 However, hypotrophy was significantly associated with cesarean delivery (16.2% vs. 5.3%; *P* = 0.001) and multiparity (23.5% vs. 2.1%; *P* = 0.001). The mean birth weight was 1883.51 ± 233.60 g. Initial Z-scores were: WAZ = 0.46 (1.15), HAZ = –0.58 (1.11), and HCZ = 1.14 (1.17). Hypotrophy was more frequent among infants born at 32–37 weeks of gestation (72.1% vs. 18.1%; *P* = 0.001). Hypotrophic infants required more phototherapy (98.1% vs. 89.7%; *P* = 0.001) and antibiotics (30.9% vs. 6.1%; *P* = 0.001).

 Exclusive breastfeeding was less frequent among hypotrophic preterm infants (94.1%) compared to their non-hypotrophic counterparts (99.5%), with a statistically significant difference (*P* = 0.002). Early introduction of water before 4 months and food diversification before 6 months were more common in hypotrophic infants (2.9% vs. 0.5% and 11.8% vs. 1.9%, respectively; *P* = 0.001). Skin-to-skin contact at birth was also less frequently practiced in hypotrophic infants (7.4% vs. 1.9%; *P* = 0.006) (Table 1).

**Table 1:** Maternal and neonatal characteristics

**Variables**	**Non-hypotrophic, n=614**		**Hypotrophic, n=72**		***P *** **value**
	**Number**	**Percent**	**Number**	**Percent**	
Maternal age (year)					0.072
< 35	593	96.0	62	91.2	
≥ 35	25	4.0	6	8.8	
Gestational age (week)					0.001
< 32	506 (	81.9	19	27.9	
32-37	112	18.1	49	72.1	
Cesarean delivery					0.001
Yes	33	5.3	11	16.2	
No	581	74.7	61	83.8	
Household income (MAD)					0.805
< 2800	611	98.9	67	98.5	
≥ 2800	7	1.1	1	1.5	
Educational levels					0.144
Primary	383	62	37	55	
Secondary and higher	235	38	31	45	
Parity					0.001
Primiparous	605	97.9	52	76.5	
Multiparous	13	2.1	16	23.5	
Phototherapy					0.001
Yes	606	98.1	61	89.7	
No	8	1.9	7	10.3	
Antibiotics use					0.001
Yes	38	6.1	21	30.9	
No	576	93.9	47	69.1	
Breastfeeding					0.002
Yes	615	99.5	64	94.1%	
No	3	0.5	4	5.9	
Early water introduction (months)					0.024
< 4	615	99.5	66	97.1	
≥ 4	3	0.5	2	2.9	
Food diversification (months)					0.001
< 6	12	1.9	8	11.8	
≥ 6	609	98.1	60	88.2	
Skin-to-skin contact					0.006
Yes	12	1.9	5	7.4	
No	602	98.1	67	92.6	

MAD = Moroccan dirham.

###  Z-Scores in hypotrophic vs non- hypotrophic preterm infants

 The analysis of Z-scores for WAZ, HAZ, and HCZ at various time points revealed significant differences between hypotrophic and non-hypotrophic newborns at each stage (*P* = 0.001 for all comparisons).

 At birth (D0), hypotrophic infants had significantly lower scores: WAZ (–1.26 (1.45) vs. 0.65 (0.93)), HAZ (–2.60 (1.16) vs. –0.36 (0.85)), and HCZ (–0.49 (1.45) vs. 1.32 (0.99)). At day 10, improvements were observed in both groups, but the gap persisted: WAZ (–0.85 (1.23) vs. 0.75 (0.81)), HAZ (–1.93 (1.36) vs. –0.02 (0.72)), and HCZ (0.27 (1.44) vs. 1.77 (0.89)).

 At 1 month, scores increased further: WAZ (0.37 (1.54) vs. 2.02 (1.58)), HAZ (–0.12 (1.47) vs. 1.46 (0.60)), and HCZ (4.01 (1.24) vs. 5.44 (0.82)). At 3 months, the trend continued: WAZ (2.29 (1.80) vs. 3.58 (1.55)), HAZ (0.55 (1.79) vs. 2.41 (1.12)), and HCZ (6.21 (2.76) vs. 7.38 (1.43)). At 6 months, although catch-up growth was evident, significant differences remained: WAZ (6.19 (2.79) vs. 8.86 (2.50)), HAZ (7.79 (1.62) vs. 9.31 (0.89)), and HCZ (7.78 (2.72) vs. 9.03 (1.88)). These findings indicate partial catch-up growth in hypotrophic infants, without complete normalization, particularly regarding linear and cranial development (Table 2).

**Table 2:** Weight for age (WAZ), Height for age (HAZ), and head circumference for age (HCZ) Z-scores according to hypotrophy status (repeated measures ANOVA)

**Z-score**	**Group**	**Mean (95% CI)**	***P *** **value**
At birth			
Weight for age	Non-hypotrophic	0.65 (0.56, 0.72)	0.001
Weight for age	Hypotrophic	–1.26 (–1.49, –1.02)	
Height for age	Non-hypotrophic	–0.36 (–0.43, –0.29)	0.001
Height for age	Hypotrophic	–2.60 (–2.80, –2.38)	
Head circumference for age	Non-hypotrophic	1.32 (1.19, 1.45)	0.001
Head circumference for age	Hypotrophic	–0.49 (–0.72, –0.25)	
10 Days			
Weight for age	Non-hypotrophic	0.75 (0.68, 0.81)	0.001
Weight for age	Hypotrophic	–0.85 (–1.05, –0.64)	
Height for age	Non-hypotrophic	–0.02 (–0.08, 0.04)	0.001
Height for age	Hypotrophic	–1.93 (–2.12, –1.74)	
Head circumference for age	Non-hypotrophic	1.77 (1.63, 1.91)	0.001
Head circumference for age	Hypotrophic	0.27 (0.03, 0.51)	
1 Month			
Weight for age	Non-hypotrophic	2.02 (1.89, 2.14)	0.001
Weight for age	Hypotrophic	0.37 (0.00, 0.74)	
Height for age	Non-hypotrophic	1.46 (1.40, 1.52)	0.001
Height for age	Hypotrophic	–0.12 (–0.29, 0.05)	
Head circumference for age	Non-hypotrophic	5.44 (5.34, 5.54)	0.001
Head circumference for age	Hypotrophic	4.01 (3.75, 4.27)	
3 Months			
Weight for age	Non-hypotrophic	3.58 (3.46, 3.71)	0.001
Weight for age	Hypotrophic	2.29 (1.90, 2.67)	
Height for age	Non-hypotrophic	2.41 (2.31, 2.51)	0.001
Height for age	Hypotrophic	0.55 (0.26, 0.84)	
Head circumference for age	Non-hypotrophic	7.38 (7.19, 7.57)	0.001
Head circumference for age	Hypotrophic	6.21 (5.77, 6.65)	
6 Months			
Weight for age	Non-hypotrophic	8.86 (8.66, 9.06)	0.001
Weight for age	Hypotrophic	6.19 (5.59, 6.79)	
Height for age	Non-hypotrophic	9.31 (9.23, 9.38)	0.001
Height for age	Hypotrophic	7.79 (7.55, 8.02)	
Head circumference for age	Non-hypotrophic	9.03 (8.87, 9.19)	0.001
Head circumference for age	Hypotrophic	7.78 (7.30, 8.25)	

###  Growth trajectories in preterm infants

**Figure 1:**
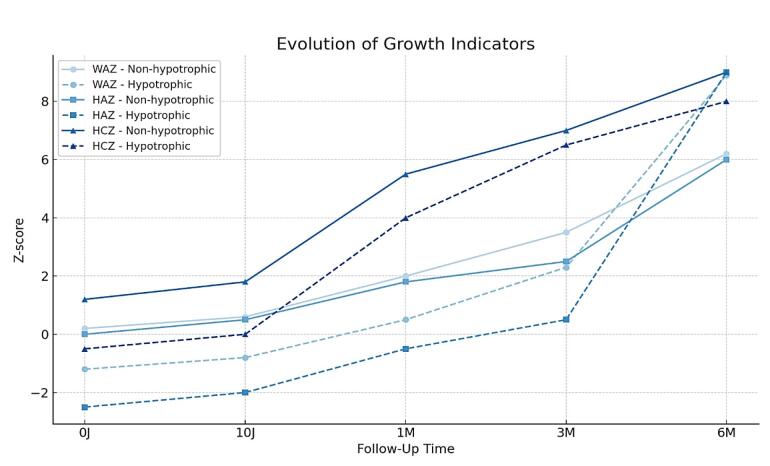
Growth trajectories from birth to 6 months in preterm infants [weight-for-age (WAZ), height-for-age (HAZ), and head circumference-for-age (HCZ) Z-scores among hypotrophic and non-hypotrophic moroccan preterm newborns at five time points: Birth (0J), 10 days (10J), 1 month (1M), 3 months (3M), and 6 months (6M)]. Hypotrophic infants showed persistently lower Z-scores across all parameters

Figure 1 illustrates the growth trajectories of weight, height, and HCZ among preterm infants based on their nutritional status at birth. Regarding WAZ, hypotrophic newborns exhibited slower growth from birth to 6 months, with an initial gap of –1.26 (1.45) vs. 0.65 (0.93). Although improvements were observed between 10 days and 3 months, they continued to lag behind at 6 months: 6.19 (2.79) vs. 8.86 (2.50), suggesting incomplete catch-up. Linear growth (HAZ) was even slower: hypotrophic infants started at –2.60 (1.16) vs. –0.36 (0.85), and despite notable progress, the gap persisted at 6 months: 7.79 (1.62) vs. 9.31 (0.89). As for HCZ, although it increased steadily, hypotrophic infants consistently remained behind their non-hypotrophic peers: –0.49 (1.45) vs. 1.32 (0.99) at birth, and 7.78 (2.72) vs. 9.03 (1.88) at 6 months, indicating incomplete cranial growth recovery. Overall, while Z-scores improved for all parameters, hypotrophic infants consistently underperformed compared to non-hypotrophic neonates throughout the follow-up period.

###  Weight catch-up and nutritional status

 Among the 686 preterm infants included in the study, the majority remained non-hypotrophic (n = 614), 67 achieved nutritional recovery, 4 experienced relapses, and only one remained hypotrophic. The proportion of infants with Z-scores < -2 progressively decreased with age. This reduction was statistically significant for WAZ (*P* < 0.001), HAZ (*P* = 0.001), and HCZ (*P* = 0.008), according to McNemar’s test. However, only 62.3% of hypotrophic neonates achieved weight catch-up (WAZ ≥ -2) by 6 months (Table 3).

**Table 3:** Change of status over time in hypotrophic and non-hypotrophic preterm infants: McNemar’s test

**Status at birth**	**Status at 6 months**	**Number**	**Percent**	***P *** **value**
Non-hypotrophic	Non-hypotrophic	614	89.5	0.001
Non-hypotrophic	Hypotrophic	4	0.6	
Hypotrophic	Non-hypotrophic	67	9.8	
Hypotrophic	Hypotrophic	1	0.1	

 Binary logistic regression identified several factors significantly associated with weight catch-up (WAZ ≥ –2) at 6 months in hypotrophic preterm infants. Table 3 presents the ORs and 95% CIs for variables associated with catch-up growth by 6 months of age. Variables included perinatal characteristics, early nutritional interventions, and hospitalization duration. Statistically significant factors (*P* < 0.05) are highlighted, while non-significant but clinically relevant trends are also indicated.

 Male gender appeared as a protective factor (OR = 0.15, 95% CI: 0.03, 0.83; *P* = 0.029), as did a hospital stay of 10 days or more (OR = 0.05, 95% CI: 0.01, 0.27; *P* = 0.001), possibly reflecting benefits from prolonged medical and nutritional support.

 Although not statistically significant, the use of antibiotics (OR = 0.18, 95% CI: 0.03, 1.01; *P* = 0.053) and early food diversification before 6 months (OR = 0.14, 95% CI: 0.02, 1.14; *P* = 0.083) showed trends toward a protective effect.

 Conversely, a gestational age of ≥ 32 weeks (OR = 6.66, 95% CI: 1.21, 36.72; *P* = 0.029) and multiparity (OR = 12.09, 95% CI: 2.12, 68.93; *P* = 0.005) were associated with an increased risk of not achieving catch-up growth (Table 4).

**Table 4:** Determinants of weight catch-up (WAZ ≥ -2) at 6 months in hypotrophic preterm infants

**Variables**	**Number**	**Percent**	**OR (95% CI)**	***P *** **value**
Phototherapy				
No	8	1.9	1.00	
Yes	667)	97.2	0.13 (0.01, 1.22)	0.075
Gender				
Female	525	76.5	1.00	
Male	161	23.5	0.15 (0.02, 0.82)	0.029
Neonatal hospitalization (day)				
< 10	37	54.4	1.00	
≥ 10	649	94.6	0.05 (0.01, 0.27)	0.001
Antibiotic use				
No	629	93.9	1.00	
Yes	59	8.6	0.18 (0.03, 1.02)	0.053
Gestational age (w)				
< 32	525	81.9	1.00	
≥ 32	161	23.5	6.66 (1.20, 36.71)	0.029
Parity				
Nuliparity	605	97.9	1.00	
Multiparity	29	4.2	12.09 (2.12, 68.92)	0.005
Food diversification (months)				
≥ 6	662	98.1	1.00	
< 6	20	2.9	0.14 (0.01, 1.30)	0.083

## Discussion

 The longitudinal analysis of our cohort reveals a marked heterogeneity in the growth trajectories of premature newborns. Our results show that hypotrophic infants have significantly lower WAZ, HAZ, and HCZ at birth and that the gap with non-hypotrophic infants persists at six months. The impact of hypotrophy at birth is particularly important. Newborns with a Z-score < -2 have a lower probability of achieving normalized growth status at six months. This is due to restricted intrauterine growth and an adverse postnatal environment due to more frequent neonatal complications (respiratory distress syndrome, infections, and delayed feeding). This observation highlights the critical importance of regular monitoring and appropriate nutritional interventions from the earliest days of life.

 This observation is consistent with several previous studies that have documented initial stunting in hypotrophic premature infants and limited catch-up capacity.^9^

 Our results show that 9.8% of hypotrophic newborns achieve a weight recovery at six months, while a minority maintain a persistent growth deficit. The analysis of the Z-scores shows that hypotrophic premature infants start with a marked deficit in weight (WAZ < -2), height (HAZ < -2), and HCZ (HCZ < -2). Catching up is a gradual but uneven process. A significant increase is observed in WAZ between birth and six months; however, hypotrophic newborns do not completely close the gap with non-hypotrophic newborns.

 The initial linear growth retardation persists at six months, reflecting an incomplete size recovery. The cranial circumference progresses slowly and remains below the non-hypotrophic average, suggesting a potential impact on neurological development.

 Although the scores increase gradually, the inability of hypotrophic newborns to reach values comparable to normal ones suggests biological and environmental limits to growth compensation. The underlying mechanisms involve metabolic alterations associated with prematurity and inadequate adaptation to the extra-uterine environment, particularly in terms of nutritional intakes and hormonal interactions such as the involvement of IGF-1 and insulin.^10^

 Our results indicate that several factors influence the weight recovery of premature babies. Gestational age, management, and parity are significant predictors of weight catch-up, which is in agreement with previous studies showing that those born at a later age and those from multiple pregnancies have better chances of recovery^11^. Our results show that most hypotrophic babies had a gestational age of 32-37 weeks. A previous study conducted by Suhag et al ^12^ suggested that being born between 32-37 weeks does not guarantee adequate fetal development and has an impact on the postpartum health and quality of life of newborns. As a result, these newborns suffer from IUGR, requiring intensive nutritional monitoring and interventions after birth.

 Guellec et al^13^ suggested that children born between 32 and 37 weeks of gestation had better recovery than those born before 32 weeks of gestation, as well as increased growth potential and fewer metabolic complications, which is in line with the results of this study.

 In addition, the link between the educational and socio-economic level of mothers and newborn growth was not significantly established, contrary to the study conducted by Rocha et al in 2021, reporting that postnatal growth was proportional to the mother’s level of education.^14^ Silva et al^15^ revealed that precarity had an influence on the development of premature infants, mainly due to the difficulty of accessing adequate nutrition and medical care.

 Hypotrophy was significantly more frequent in multiparous women (*P* < 0.001) and in twin pregnancies (*P* = 0.032). This result is consistent with previous studies showing that high parity is a risk factor for IUGR due to depletion of maternal reserves.^16^ This result is in agreement with the study of Boghossian et al, who confirmed that multiparous pregnancies were associated with intrauterine growth retardation and postpartum complications.^11^ We observed a significantly higher number of caesarean sections in hypotrophic newborns. This is in agreement with the study conducted by Cao et al^17^ and may be linked to the fact that caesarean section is often performed in cases of fetal distress or severe intrauterine growth retardation.

 According to our study, the exclusive breastfeeding rate is significantly lower in individuals suffering from hypotrophy, amounting to 94.1%, vs 99.5% in non-hypotrophic newborns (*P* = 0.002). This result concurs with that of the study conducted by Horta et al in 2023^18^ and can be explained by the sucking and feeding problems encountered by fragile infants, requiring the implementation of a special protocol for this vulnerable population. Several studies confirmed that breastfeeding is the healthiest choice for newborn and infant nutrition. Breastfeeding is associated with a reduced risk of mortality^19^ as it provides several bioactive molecules that contribute to immune maturation, organ development, and healthy gut microbial colonization, ensuring an appropriate immunological response that protects the newborn from infection and inflammation from birth.^20^

 In addition, Demirci^21^ proved that breastfeeding also helps reduce the risk of developing long-term metabolic disorders, underlining the importance of this practice for the overall health of infants. Starting solid foods before six months was significantly more common in hypotrophic newborns than in non-hypotrophic infants (11.8% vs 1.9%) (*P* < 0.001). These results suggest the possibility of nutritional compensation in these hypotrophic premature newborns, which is in line with the study conducted by Fewtrell et al, who support international guidelines of introducing solids at 6 months for best health outcomes.^22^

 We found a correlation between stunting and starting a diversified diet before six months, which is in agreement with previous studies showing that this early practice in premature babies can lead to harmful consequences on the growth of premature babies, including their immature digestive system and the risk of contracting infectious diseases due to low immunity in this population. It can also lead to a decrease in the intake of essential calories and proteins during this crucial phase of development.^23^

 The persistence of differences between groups suggests that other factors, including genetic and epigenetic factors, may influence the weight recovery of hypotrophic premature infants. A multidisciplinary approach integrating early nutritional interventions and more in-depth longitudinal monitoring could improve the growth trajectories of these vulnerable infants. The impact of neonatal hospitalization on weight recovery is a notable result. Prolonged hospitalization may reflect increased monitoring and optimized nutritional support; however, other studies have shown that nosocomial infections and hospital stress can impair growth.^24^

 An interesting result is the negative effect of antibiotics on weight recovery. The use of antibiotics in the neonatal period has been associated with altered gut microbiota, which may affect nutrient absorption and metabolic development.^25^ In addition, the vulnerability of hypotrophic newborns requires more treatments such as phototherapy and early antibiotic therapy, making them more susceptible to infections and metabolic complications.

 In 2024, Wang et al^26^ confirmed that the early use of antibiotics could disrupt the balance of the intestinal microbiota and thus hinder the growth of newborns after birth. This significant exposure may be associated with hyperbilirubinemia that is commonly seen in premature infants with IUGR.

 Eghbalian et al^27^ also highlighted a link between hyperbilirubinemia and stunting in premature infants and suggested that early antibiotic administration may lead to changes in the composition of the gut microbiota and can impair their ability to absorb nutrients. The earlier and more intensive medical care and the close monitoring of bilirubin in premature infants with in vitro fertilization (IVF) are of great importance.

 Our study agrees with the study conducted by Chu et al,^28^ indicating that most premature babies with low birth weight have the ability to catch up in their first year of life, especially when they are in a nutritionally beneficial environment. Nevertheless, a significant number of hypotrophic newborns did not reach growth levels similar to those of non-hypotrophic children in our study.

 The findings of this study highlight several clinical and perinatal factors associated with incomplete weight catch-up in hypotrophic preterm infants. From a clinical perspective, these results emphasize the importance of intensive nutritional monitoring during the first six months, especially in high-risk newborns. The positive association between prolonged hospitalization and improved catch-up suggests that intensive inpatient care, including controlled enteral and parenteral nutrition, may play a crucial role in enhancing postnatal growth. Furthermore, the lower frequency of exclusive breastfeeding and the early introduction of water or complementary foods observed in hypotrophic infants indicate suboptimal feeding practices, which can negatively affect growth outcomes. These observations underscore the need for strengthened parental education and early intervention strategies to promote appropriate feeding practices. Lastly, the persistence of significant Z-score differences at six months, despite partial catch-up, suggests the necessity for extended monitoring beyond this period, particularly for linear growth and cranial growth which are considered key indicators of future neurocognitive development.

 This single-center study conducted in Rabat limits the generalizability of the findings. The small sample size of hypotrophic infants (n = 72) reduces statistical power for subgroup analyses. Measurement errors or inter-observer variability may have occurred. Finally, the lack of follow-up beyond six months prevents assessment of long-term growth and developmental outcomes.

**Highlights**

- This study was conducted on 686 preterm infants in Morocco and explored postnatal growth trajectories using WHO standards and Fenton 2013 Z-scores.
- 10.5% of Moroccan preterm infants were hypotrophic at birth (Z-score < -2).
- Only 62.3% of hypotrophic infants achieved weight catch-up by 6 months, with residual deficits.
- Male gender and neonatal hospitalization of ≥ 10 days were significant predictors of weight recovery.
- The results underline the urgent interventions and longitudinal follow-up of vulnerable neonatal populations in low- and middle-income countries.

## Conclusion

 Although partial catch-up growth was observed in many preterm infants, significant growth gaps remained at six months, particularly among hypotrophic newborns. These findings highlight the need for sustained multidisciplinary strategies, including early nutritional support, careful introduction of solid foods, standardized growth monitoring, and prudent medical management, to optimize long-term outcomes in this vulnerable population.

 This study demonstrated that among Moroccan preterm infants, those born hypotrophic (Z-score < –2) exhibited significantly impaired growth trajectories compared to their non-hypotrophic counterparts, across all anthropometric indicators including WAZ, HAZ, and HCZ from birth to six months. While partial catch-up growth was observed in the hypotrophic group, particularly in weight, complete normalization was not achieved in linear or cranial growth.

 The logistic regression analysis identified key predictors of incomplete weight catch-up at six months, including shorter neonatal hospitalization and female gender, whereas multiparity and gestational age of ≥ 32 weeks were paradoxically associated with persistent growth restriction. Additionally, suboptimal feeding practices such as early food diversification and reduced exclusive breastfeeding rates were more frequent among hypotrophic infants, potentially contributing to their delayed growth recovery.

 These findings suggest that growth restriction at birth has lasting effects through early infancy and that catch-up growth in hypotrophic preterm infants is both incomplete and heterogeneous. Targeted interventions, such as prolonged hospital-based nutritional support, exclusive breastfeeding promotion, and delayed introduction of complementary foods, are needed to improve postnatal growth outcomes in this high-risk population. Furthermore, the persistent gaps in HAZ and HCZ underscore the need for long-term neurodevelopmental monitoring beyond the six-month period.

## Perspectives

- Long-term follow-up should be extended to school age to assess potential neurodevelopmental complications.
- Early nutritional strategies should be developed to prevent metabolic diseases in later life.
- Different feeding protocols (exclusive breastfeeding vs. mixed feeding) should be compared to determine the most effective approach.

## Acknowledgements

 The authors would like to thank the National Reference Center for Neonatology and Nutrition in Rabat for logistical support and data access, as well as the Biomedical Research Ethics Committee of Mohammed V University in Rabat for protocol evaluation. We extend our gratitude to the medical and nursing personnel for their rigorous anthropometric data collection.

## Competing Interests

 The authors declare that they have no competing interests.

## Ethical Approval

 This retrospective study was based on anonymized data extracted from electronic medical records, with no direct contact with participants. In accordance with university hospital practices, general consent for the use of medical data for research purposes is systematically obtained from parents or guardians at admission. The protocol was approved by the Biomedical Research Ethics Committee of Mohammed V University of Rabat (reference: C64/20), in compliance with the Declaration of Helsinki and national data protection regulations.

## Funding

 None.
